# Clinical coding of long Covid in Wales: A cohort study of 3.5 million people using linked health and demographic data

**DOI:** 10.23889/ijpds.v8i2.2308

**Published:** 2023-09-14

**Authors:** Hoda Abbasizanjani, Stuart Bedston, Lucy Robinson, Matthew Curds, Ashley Akbari

**Affiliations:** 1 Population Data Science, Swansea University Medical School, Faculty of Medicine, Health and Life Science, Swansea University, Swansea, United Kingdom; 2 Welsh Government, Cardiff, United Kingdom

## Objectives

‘Long COVID’ (LC) is broadly defined as signs and symptoms that continue or develop after the acute phase of COVID-19, and can affect cardiovascular, respiratory and other organ systems. Using electronic health records, we investigated clinical coding of LC in primary and secondary care for the population of Wales.

## Methods

We conducted a cohort study for the population of Wales, using anonymised individual-level linked data in the Secure Anonymised Information Linkage (SAIL) Databank. We used the Welsh COVID-19 e-cohort (doi:10.1136/bmjopen-2020-043010), which consists of all people (adults and children) alive and resident in Wales from 1st January 2020. To this e-cohort we linked primary and secondary care, COVID-19 testing, and ethnic group data. We then calculated the proportion of people with a LC diagnosis code (in primary and secondary care data) overall and stratified by demographic variables.

## Results

Of 3.5m residents, 7,696 (0.2%) had a LC clinical diagnosis. Compared with the general population, a higher proportion of people with LC were female, middle age, white, and hospitalised within 28 days of a confirmed COVID-19 infection. LC affected all socioeconomic groups, as assessed using the Welsh Index of Multiple Deprivation. When looking at LC diagnosis codes in primary care, 30.9% of practices in SAIL have not used these codes at all. And the number of recorded events was low until the end of January 2021, after which there was an increase in coding. These findings are likely a substantial underestimate of LC prevalence in Wales. Earlier estimates from self-reported surveys, such as the Office for National Statistics, are much higher, ranging anywhere between 3-5%.

## Conclusion

Low recording rates of LC and variation between practices could be due to a delay in introducing clinical coding and lack of presentation/recording. Understanding prevalence of LC is vital for addressing the scale of the problem. Therefore developing additional data-driven approaches is necessary to obtain an accurate prevalence estimate.

